# *Eisenia bicyclis*-Mediated Gold Nanoparticles Exhibit Antibiofilm and Antivirulence Activities Against *Pseudomonas aeruginosa* and *Staphylococcus aureus*

**DOI:** 10.3390/antibiotics14020182

**Published:** 2025-02-11

**Authors:** Do Kyung Oh, Du-Min Jo, Nam-Gyun Kim, Kyung-Jin Cho, Geum-Jae Jeong, Nazia Tabassum, Won-Kyo Jung, Fazlurrahman Khan, Young-Mog Kim

**Affiliations:** 1 Department of Food Science and Technology, Pukyong National University, Busan 48513, Republic of Korea; dkoh@pknu.ac.kr (D.K.O.); rudwls803342@gmail.com (K.-J.C.); jgj1994@pukyong.ac.kr (G.-J.J.); 2 Marine Integrated Biomedical Technology Center, The National Key Research Institutes in Universities, Pukyong National University, Busan 48513, Republic of Korea; amagin12@naver.com (N.-G.K.); nazia99@pukyong.ac.kr (N.T.); wkjung@pknu.ac.kr (W.-K.J.); 3 Research Center for Marine Integrated Bionics Technology, Pukyong National University, Busan 48513, Republic of Korea; 4 National Marine Biodiversity of Korea (MABIK), Seochun 33662, Republic of Korea; dmjo@mabik.re.kr; 5 Major of Biomedical Engineering, Division of Smart Healthcare, College of Information Technology and Convergence and New-Senior Healthcare Innovation Center (BK21 Plus), Pukyong National University, Busan 48513, Republic of Korea; 6 Ocean and Fisheries Development International Cooperation Institute, Pukyong National University, Busan 48513, Republic of Korea; 7 International Graduate Program of Fisheries Science, Pukyong National University, Busan 48513, Republic of Korea

**Keywords:** *Eisenia bicyclis*, gold nanoparticles, antibiofilm, antivirulence, secondary metabolites, gene expression

## Abstract

**Background/Objectives:** Brown algae, particularly *Eisenia bicyclis*, produce various bioactive chemicals with significant application potential in the food, cosmetics, and pharmaceutical industries. This study aimed to evaluate the antibacterial, antibiofilm, and antivirulence properties of the ethyl acetate fraction (EA) of *E. bicyclis* and its synthesized gold nanoparticles (EA-AuNPs), with a focus on their potential applications against both Gram-positive and Gram-negative bacteria. **Methods:** The bioactive component in the ethyl acetate fraction was identified using a gas chromatography-mass spectroscopy (GC-MS) device and a liquid chromatography-mass spectrometer/mass spectrometry (LC-MS) system. The crystal violet method was utilized to evaluate the biofilm inhibition experiments. Several instruments, including dynamic light scattering, Fourier transform infrared, X-ray diffraction, field emission transmission electron microscopy, and energy-dispersive spectroscopy, were employed to completely characterize the produced EA-AuNPs. The cytotoxicity of the EA-AuNPs was determined using the MTT assay, and the expression of genes linked with biofilm and virulence in *Pseudomonas aeruginosa* and *Staphylococcus aureus* was investigated using real-time polymerase chain reaction (RT-PCR). **Results:** Various bioactive compounds were identified from the EA using GC-MS and LC-MS, including fatty acids and phlorotannins such as eckol, dieckol, 6,6’-bieckol, and phlorofucofuroeckol in high amounts, highlighting EA as a phlorotannin-rich fraction. The EA also demonstrated significant antibiofilm activity, with 79.86% inhibition at 512 μg/mL against *P. aeruginosa* and 87.00% at 64 μg/mL against *S. aureus*. EA was then used in the synthesis of gold nanoparticles (AuNPs) to improve their stability and safety. The synthesized EA-AuNPs were determined to have an average size of 165.04 nm, with a zeta potential of −29.86 mV, indicating good stability. In antibiofilm activity assays, EA-AuNPs demonstrated 45.76% inhibition against *P. aeruginosa* at 1024 μg/mL and 44.64% inhibition against *S. aureus* at 128 μg/mL. At sub-MIC levels, EA-AuNPs significantly inhibited biofilm formation and virulence factors, including the motility of *P. aeruginosa* and staphyloxanthin synthesis in *S. aureus*. The RT-PCR analysis revealed the downregulation of key genes involved in biofilm formation and virulence in *P. aeruginosa* and *S. aureus*. **Conclusions:** These findings highlight the potential of *E. bicyclis* solvent-soluble extracts and EA-AuNPs as effective antibacterial, antibiofilm, and antivirulence agents, with significant application potential in the pharmaceutical and food industries. To the best of our knowledge, this is the first report of antibiofilm activity against both Gram-positive and Gram-negative bacteria using EA-AuNPs.

## 1. Introduction

*Pseudomonas aeruginosa* and *Staphylococcus aureus* are significant pathogens in hospital-acquired infections, particularly in immunocompromised individuals and cystic fibrosis patients [1,2]. Both exhibit multiple antibiotic resistance mechanisms, including efflux pumps, β-lactamases, and outer membrane modifications, and contribute to chronic infections through virulence factors and biofilm formation [3,4]. Their ability to modulate host immune responses and adapt to the airway environment further complicates treatment [5]. In cystic fibrosis, the interaction between *P. aeruginosa* and *S. aureus* is complex, involving both competition and cooperation, which impacts patient outcomes [6]. *Staphylococcus aureus*, especially methicillin-resistant strains (MRSA), poses a global health threat due to its increasing resistance and diverse virulence factors [7]. Both *P. aeruginosa* and *S. aureus* have been recognized as priority pathogens by the World Health Organization (WHO) [8].

The need for innovative treatment approaches has led to the development of inhaled antibiotics, targeting chronic pulmonary infections with higher airway concentrations and reduced systemic toxicity [9]. Additionally, novel strategies targeting virulence factors and antibiotic resistance are being explored, including bacteriophage-derived antimicrobials, antivirulence treatments, and nanoparticle-based drug delivery systems [10,11]. Innovative therapies such as disrupting biofilms, inhibiting toxin production, and using combination antibody therapies show promise for combating these pathogens [11,12]. Recent studies have explored the green synthesis of gold nanoparticles (AuNPs) using marine algae extracts, including *Eisenia bicyclis*, as an eco-friendly alternative to traditional methods [13,14]. *Eisenia bicyclis*, a brown alga, demonstrates significant antimicrobial, antibiofilm, and antivirulence properties. Its extracts, particularly phlorotannins like dieckol and phlorofucofuroeckol-A, exhibit antiviral activity against murine norovirus [15] and antibacterial effects against acne-related bacteria [16] and MRSA [17]. These compounds also show synergistic effects with antibiotics [16,17].

The biosynthesized AuNPs exhibit unique properties and have shown promising antimicrobial activity against various pathogens [18,19]. The algal extracts contain reducing and capping agents, such as polyphenols and carbohydrates, which facilitate AuNP formation and stabilization [20]. The resulting AuNPs are typically spherical, with sizes ranging from 6 to 35 nm, and demonstrate good stability [18]. In addition to their antimicrobial properties, algae-mediated AuNPs have shown potential in catalysis, anti-inflammatory applications, and cancer treatment [19]. This green synthesis approach offers a simple, cost-effective, and environmentally friendly method for producing AuNPs with diverse biomedical applications [21]. AuNPs possess unique properties, such as high surface area and the ability to interact with bacterial membranes, contributing to their antibacterial effects [22]. They can disrupt bacterial cell membranes, interfere with DNA and protein synthesis, and induce oxidative stress, thereby inhibiting bacterial growth [23]. AuNPs synthesized from marine algae, such as *Undaria pinnatifida*, have demonstrated effectiveness against biofilm-producing bacteria and offer a cost-effective and sustainable alternative to traditional antibiotics [18]. Similarly, a recent study found that AuNPs generated from the methanol (MeOH) extract of *Eisenia bicyclis* have antibiofilm and antivirulence activity against *P. aeruginosa* and *S. aureus* [24]; however, these effects were observed at the phenotypic level.

In the present study, we identified bioactive compounds from the ethyl acetate extract (EA) of *E. bicyclis*, synthesized EA-gold nanoparticles (EA-AuNPs), and characterized their antibiofilm effects at the phenotypic level. The effects were validated by expressing biofilm- and virulence-associated genes in *P. aeruginosa* and *S. aureus*. The study was organized into the following key steps: (i) isolation and identification of bioactive metabolites from EA fractions, (ii) synthesis and characterization of EA-AuNPs using EA, (iii) determination of the minimum inhibitory concentrations of EA and EA-AuNPs, (iv) study of the antibiofilm effect of EA and EA-AuNPs, (v) assessment of the cytotoxicity of EA and EA-AuNPs on animal cell cultures, (vi) evaluation of the virulence-inhibitory effects of EA and EA-AuNPs, and (vii) examination of the expression of genes associated with biofilm formation and virulence.

## 2. Results

### 2.1. Isolation and Identification of Bioactive Metabolites from EA

**Figure 1:**
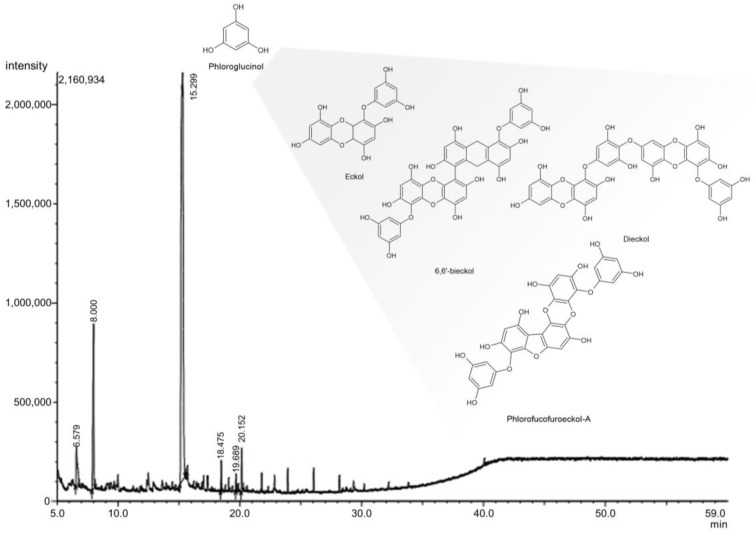
Bioactive compounds identified in *Eisenia bicyclis* ethyl acetate fraction by GC-MS and LC-MS/MS analyses. Phloroglucinol was identified in GC-MS (at the RT: 15.299 min), whereas the other compounds present in the shadowed area were identified by LC-MS/MS analysis.

GC-MS analysis of the EA, prepared through liquid–liquid extraction (Figure S1), revealed six distinct compounds (Table S3). Phloroglucinol was the predominant component, constituting 78.58% of the fraction. Other identified compounds included succinic acid (12.24%), succinic anhydride (5.11%), palmitic acid (1.97%), and neophytic dienes (1.22%). In contrast, the MeOH extract contained various fatty acids but no detectable phloroglucinol. LC-MS/MS analysis also revealed the presence of phlorotannin in the EA (Figure 1). Eckol, dieckol, 6,6’-bieckol, and phlorofucofuroeckol were identified (Table S4).

### 2.2. Synthesis and Characterization of EA-AuNPs Using EA

**Figure 2:**
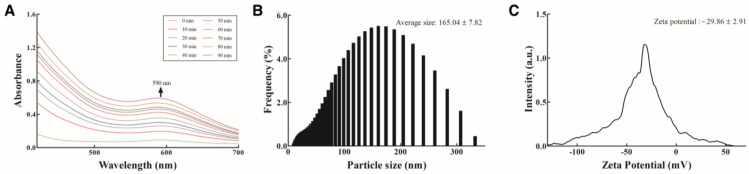
Physiochemical characteristics of *Eisenia bicyclis* ethyl acetate fraction conjugated with gold nanoparticles (EA-AuNPs). (**A**) UV–visible absorption spectra, (**B**) size distribution of EA-AuNPs, and (**C**) zeta potential of EA-AuNPs.

EA-AuNPs were synthesized and characterized as described in the Materials and Methods section. The synthesized EA-AuNPs exhibited a maximum absorbance peak at 590 nm (Figure 2A), which corresponds to the localized surface plasmon resonance (LSPR) property of AuNPs, typically observed within the range of 500–600 nm [25]. The average size of the EA-AuNPs was 165.04 ± 7.82 nm (Figure 2B). The zeta potential of the synthesized EA-AuNPs was measured to assess their stability and surface charge, yielding a value of −29.86 ± 2.91 mV (Figure 2C).

**Figure 3:**
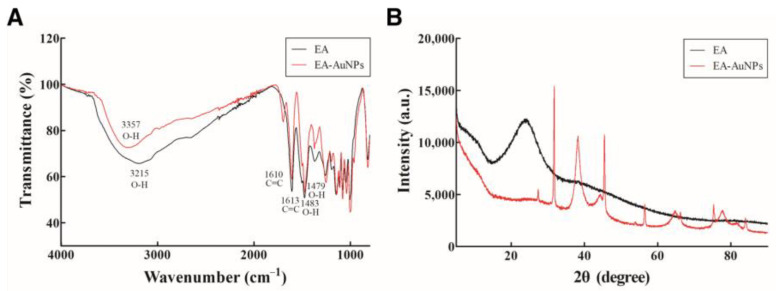
Physiochemical characterization of *Eisenia bicyclis* ethyl acetate fraction conjugated with gold nanoparticles (EA-AuNPs). (**A**) FTIR spectra of EA and EA-AuNPs. (**B**) XRD spectra of *Eisenia bicyclis* ethyl acetate fraction (EA) and EA-AuNPs.

Surface characterization of EA and EA-AuNPs via FT-IR analysis revealed broad peaks at 3357 cm^−1^ and 3215 cm^−1^, which were likely associated with -OH stretching in hydroxyl groups from polyphenols and polysaccharides in *E. bicyclis* (Figure 3A). The presence of C=C bands in both EA and EA-AuNPs was observed at 1696 cm^−1^, 1610 cm^−1^, and 1612 cm^−1^, indicative of conjugated alkenes. The region between 1084 and 1270 cm^−1^ was attributed to the C-O group. The interaction between Au^+^ ions and EA metabolites was marked by a decrease in relative intensities, suggesting capping and stabilization of the nanoparticles, a trend that aligns with previous findings [26].

XRD pattern analysis was employed to confirm the crystalline structure of the synthesized EA-AuNPs, which exhibited distinct peaks at 2θ values of 38.10°, 45.45°, 64.09°, and 77.11° (Figure 3B). These diffraction peaks correspond to the characteristic crystallinity profile and thus confirm the crystalline nature of AuNPs [27,28]. In contrast, the EA displayed an amorphous peak at 23.20° (Figure 3B). The XRD pattern analysis of EA-AuNPs is consistent with observations from previous studies [29].

**Figure 4:**
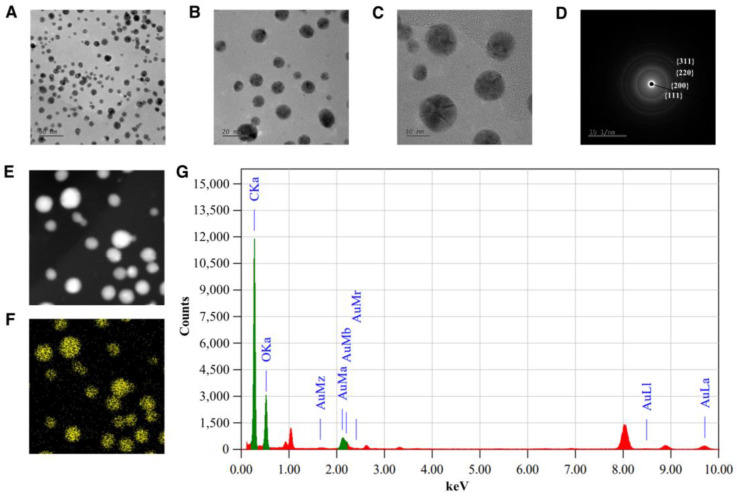
Morphological and elemental analysis of *Eisenia bicyclis* ethyl acetate fraction conjugated with gold nanoparticles (EA-AuNPs) using FE-TEM and EDS. FE-TEM image at (**A**) 50 nm resolution, (**B**) 20 nm resolution, (**C**) 10 nm resolution; (**D**) SAED of EA-AuNPs; (**E**) SEM image of EA-AuNPs; (**F**) mapping of Au element; and (**G**) EDS spectra of EA-AuNPs.

The morphology of the EA-AuNPs was also analyzed using FE-TEM, which confirmed that the nanoparticles predominantly exhibited a spherical shape (Figure 4A–C). SAED analysis was used to verify the crystallinity and structural properties of the synthesized AuNPs. The SAED pattern exhibited distinct diffraction rings, corresponding to the characteristic planes of the face-centered cubic (FCC) structure of gold, such as {111}, {200}, and {220} (Figure 4D). Additionally, the {311} plane of the FCC structure of gold nanoparticles corresponded to the XRD peak at 38.10°, indicating the high crystallinity and structural purity of the synthesized AuNPs. This finding confirms the successful synthesis of highly crystalline and polycrystalline gold nanoparticles, which are essential for their stability and functional performance.

EDS was performed to verify the elemental composition of the synthesized nanoparticles (Figure 4G). Elemental mapping also validated the presence of Au within the nanoparticles (Figure 4F). SEM, complemented by EDS analysis, further confirmed that the EA-AuNPs exhibited a spherical morphology, consistent with FE-TEM observations (Figure 4E).

These findings confirmed the successful synthesis of EA-AuNPs using the EA. Consequently, antibacterial and antibiofilm assays were performed to evaluate the bioactive properties of the EA and EA-AuNPs.

### 2.3. Minimum Inhibitory Concentrations of E. bicyclis Solvent-Soluble Fractions and EA-AuNPs

The MIC results of *E. bicyclis* solvent-soluble fractions are shown in Table 1. Among the *E. bicyclis* solvent-soluble fractions, only EA exhibited antibacterial activity against *P. aeruginosa* at 1024 μg/mL. MeOH, DCM, *n*-hexane fractions, and EA exhibited higher antibacterial activities against *S. aureus*, with EA showing significant activities at 128 μg/mL. In a recent study, we found that the MeOH extract (EB) and EB-AuNPs have broad-spectrum antibacterial activity against bacterial pathogens [24].

Thus, the *E. bicyclis* fractions were found to exert higher antibacterial activity against *S. aureus* than *P. aeruginosa*, with the EA showing the highest activity. Synthesized EA-AuNPs were also shown to have antibacterial activity at 2048 μg/mL against *P. aeruginosa* and 256 μg/mL against *S. aureus*.

### 2.4. Biofilm Inhibition Effect of E. bicyclis Solvent-Soluble Fractions and EA-AuNPs

**Figure 5:**
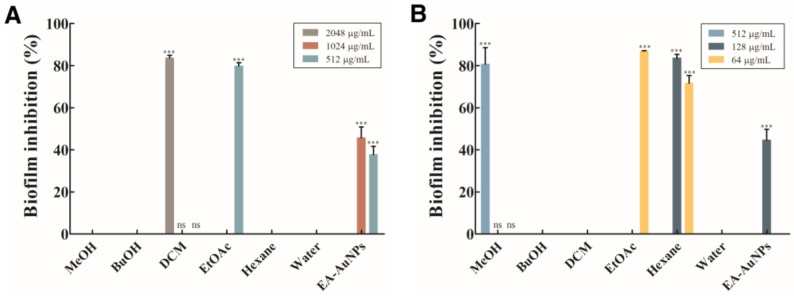
Biofilm inhibitory effect of *Eisenia bicyclis* solvent-soluble fractions and *Eisenia bicyclis* ethyl acetate fraction conjugated with gold nanoparticles (EA-AuNPs). (**A**) *Pseudomonas aeruginosa* and (**B**) *Staphylococcus aureus*. *** Denotes significant difference at *p* < 0.001; ns denotes no significance.

The biofilm inhibitory activities of *E. bicyclis* solvent-soluble fractions and EA-AuNPs are shown in Figure 5. Among the Gram-negative bacteria, *P. aeruginosa* was significantly inhibited by the DCM fraction and EA. The DCM fraction showed 83.69% inhibition at 2048 μg/mL, whereas the EA showed 79.86% inhibition at 512 μg/mL against *P. aeruginosa*. EA-AuNPs demonstrated an inhibition rate of 45.76% at 1024 μg/mL. The Gram-positive *S. aureus* biofilm was inhibited by the MeOH fraction at a rate of 80.75% at 512 μg/mL concentration, the EA at 87.00% at 64 μg/mL, and the *n*-hexane fraction at 83.67% at 128 μg/mL. EA-AuNPs exhibited a 44.64% inhibition rate at 128 μg/mL. Thus, the EA displayed the highest inhibition levels among the *E. bicyclis* solvent-soluble fractions, in line with the MIC results. Moreover, the highest biofilm inhibition activity against *S. aureus* was obtained with EA-AuNPs.

In this study, EA and EA-AuNPs exhibited significant inhibitory activity against biofilms formed by pathogenic microorganisms. The seaweed-derived nanoparticles used in this study were shown to inhibit biofilms formed by Gram-positive as well as Gram-negative bacteria. The binding of components of *E. bicyclis* extracts to metal conjugates demonstrated superior antibacterial and antibiofilm activities.

### 2.5. SEM Analysis of Biofilm Inhibition of EA and EA-AuNPs Against Pathogenic Bacteria

Mature biofilms of Gram-negative *P. aeruginosa* and Gram-positive *S. aureus* were treated with sub-MIC concentrations of EA and EA-AuNPs. SEM images of the treated biofilms are shown in Figure S2. Both *P. aeruginosa* and *S. aureus* exhibited reduced cell aggregation and biofilm formation, with decreased bacterial colonization observed in samples treated with EA and EA-AuNPs compared to the controls.

### 2.6. Cytotoxicity Assessment of EA and EA-AuNPs on RAW 264.7 Cells

**Figure 6:**
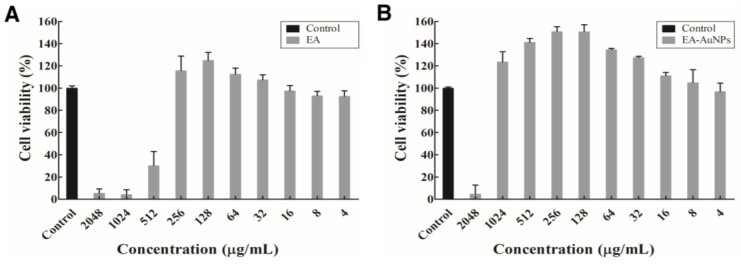
Cytotoxic effects of *Eisenia bicyclis* ethyl acetate fraction (EA) and EA conjugated with gold nanoparticles (EA-AuNPs) in RAW 264.7 cells. (**A**) EA and (**B**) EA-AuNPs.

The Cell Counting Kit-8 (CCK-8) test was used to examine the cytotoxic effects of EA and EA-AuNPs (Figure 6). Cell viability was 4.11–30.19% following EA treatment at concentrations of 512–2048 µg/mL. Below this concentration range, no cytotoxicity was observed. EA-AuNPs reduced cell viability to 4.54% ± 8.20% at 2048 µg/mL, whereas other concentrations did not exhibit any toxicity. Based on these findings, EA-AuNPs were considered safer than other forms of extracts of *E. bicyclis*.

### 2.7. Anti-Motility Effect of EA and EA-AuNPs on P. aeruginosa

**Figure 7:**
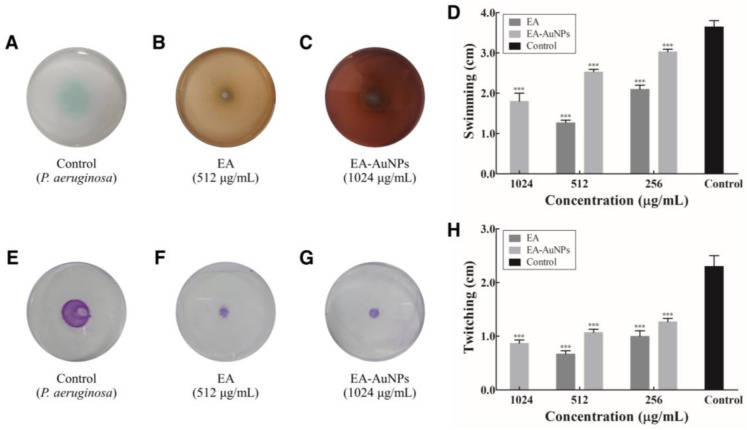
Attenuation of *Pseudomonas aeruginosa* motility by *Eisenia bicyclis* ethyl acetate fraction (EA) and EA conjugated with gold nanoparticles (EA-AuNPs). (**A**) Swimming motility of control cells. (**B**) Swimming motility in the presence of EA. (**C**) Swimming motility in the presence of EA-AuNPs. (**D**) Bar graph showing the percentage of swimming inhibition. (**E**) Twitching motility of control cells. (**F**) Twitching motility in the presence of EA. (**G**) Twitching motility in the presence of EA-AuNPs. (**H**) Graph showing the percentage of twitching inhibition. *** Denotes significant difference at *p* < 0.001.

*P. aeruginosa* is a Gram-negative bacterium with flagella that allows it to migrate toward biotic or abiotic surfaces and initiate biofilm formation. Figure 7 illustrates the impact of EA and EA-AuNPs on the motility of *P. aeruginosa*, specifically the movement mediated by flagella and pili. Swimming motility was inhibited by 42.47–65.30% with EA treatment at 256–512 μg/mL and by 16.89–50.68% with EA-AuNPs treatment at 256–1024 μg/mL compared to the control group. Twitching motility is a surface movement regulated by the extension and retraction of the pili. Both EA and EA-AuNPs significantly suppressed this type of movement. Compared to the control group, EA treatment at 256–512 µg/mL inhibited twitching by 56.52–71.01%, whereas EA-AuNPs treatment at 256–1024 µg/mL showed an inhibition rate of 44.93–62.32%. Although EA exhibited a more significant effect, our findings demonstrated that both samples had outstanding twitching motility inhibition activity.

### 2.8. Attenuation of P. aeruginosa Virulence upon EA and EA-AuNPs Treatment

**Figure 8:**
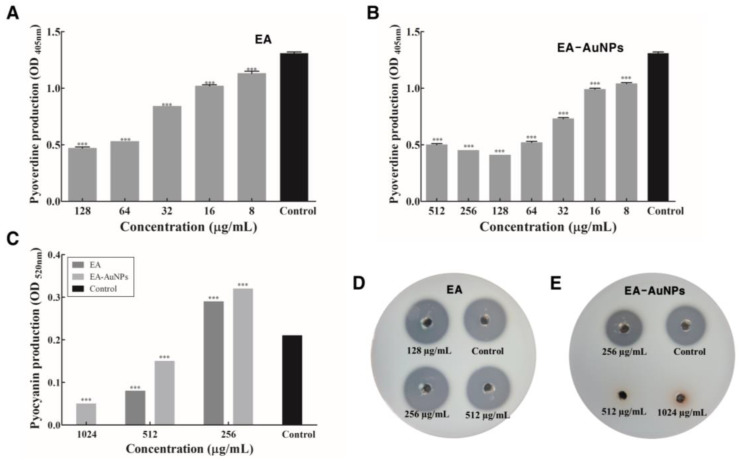
Inhibition of virulence factors in *Pseudomonas aeruginosa* by *Eisenia bicyclis* ethyl acetate fraction (EA) and EA conjugated with gold nanoparticles (EA-AuNPs). (**A**) Pyoverdine production in the presence of EA. (**B**) Pyoverdine production in the presence of EA-AuNPs. (**C**) Pyocyanin production in the presence of EA and EA-AuNPs. (**D**) Protease inhibition by EA at concentrations ranging from 256 to 1024 μg/mL. (**E**) Protease inhibition by EA-AuNPs at concentrations ranging from 256 to 1024 μg/mL. *** Denotes significant difference at *p* < 0.001.

EA inhibited pyoverdine by 13.59–64.37% following treatment with 8 to 128 µg/mL, while EA-AuNPs demonstrated inhibition rates ranging from 68.79% to 24.09% at concentrations between 8 and 512 µg/mL (Figure 8A,B). EA (512 µg/mL) and EA-AuNPs (1024 µg/mL) exhibited substantial inhibitory effects on pyocyanin production, with 60.44% and 77.91% inhibition rates, respectively (Figure 8C). EA-AuNPs were found to completely inhibit protease production at concentrations above 512 µg/mL, with significant reductions observed even at 256 µg/mL compared to the control (Figure 8E). In contrast, EA did not inhibit protease production at any concentration (Figure 8D). EA and EA-AuNPs also inhibited pyoverdine, pyocyanin, and protease, which are virulence factors produced by *P. aeruginosa* (Figure 8).

### 2.9. Inhibition of Staphyloxanthin Production upon Treatment with EA and EA-AuNPs in S. aureus

**Figure 9:**
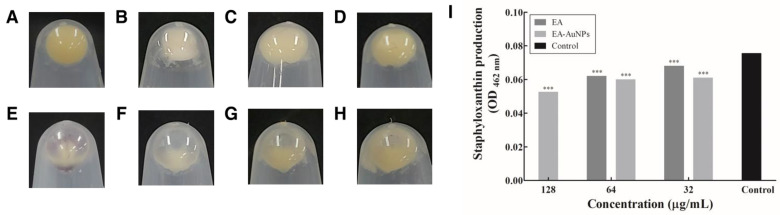
Suppression of staphyloxanthin production in *Staphylococcus aureus* by *Eisenia bicyclis* ethyl acetate fraction (EA) and EA conjugated with gold nanoparticles (EA-AuNPs). (**A**) Untreated control cells. (**B**) Cells treated with EA at 64 μg/mL. (**C**) Cells treated with EA at 32 μg/mL. (**D**) Cells treated with EA at 16 μg/mL. (**E**) Cells treated with EA-AuNPs at 128 μg/mL. (**F**) Cells treated with EA-AuNPs at 64 μg/mL. (**G**) Cells treated with EA-AuNPs at 32 μg/mL. (**H**) Cells treated with EA-AuNPs at 16 μg/mL. (**I**) Quantification of staphyloxanthin production using spectrophotometric analysis. *** Denotes significant difference at *p* < 0.001.

*S. aureus* cell pellets displayed a distinct yellow color corresponding to the presence of staphyloxanthin (Figure 9). Upon treatment with increasing concentrations of EA and EA-AuNPs, the golden-yellow color gradually faded to white, indicating pigment inhibition. In the staphyloxanthin extraction assay, EA demonstrated 17.88% inhibition at 64 µg/mL, whereas EA-AuNPs achieved a higher inhibition rate of 30.46% at 128 µg/mL.

### 2.10. EA and EA-AuNPs Suppress the Genes Associated with Biofilm and Virulence Factors

**Figure 10:**
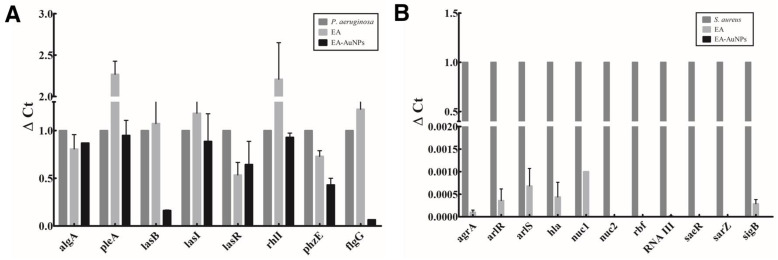
Relative expression levels of genes associated with biofilm formation and virulence following treatment with *Eisenia bicyclis* ethyl acetate fraction (EA) and EA conjugated with gold nanoparticles (EA-AuNPs). (**A**) *Pseudomonas aeruginosa* and (**B**) *Staphylococcus aureus*.

RT-PCR analysis demonstrated that both EA and EA-AuNPs effectively inhibited the expression of genes involved in biofilm formation and virulence factor production in *P. aeruginosa* and *S. aureus* at sub-MICs (Figure 10). Specifically, *P. aeruginosa* was treated with EA at 512 µg/mL and EA-AuNPs at 1024 µg/mL, while *S. aureus* was treated with EA at 64 µg/mL and EA-AuNPs at 128 µg/mL. In *P. aeruginosa*, genes encoding elastase (*lasB*), QS-related genes (*lasR*), gene encoding anthranilate synthase (*phzE*), and motility-related factor (*flgG*) showed significant suppression of gene expression, with EA-AuNPs achieving 5.15–93.4% inhibition, whereas EA alone exhibited only modest effects. Similarly, genes associated with biofilm formation (*rbf*) and QS regulation [30] were significantly downregulated in *S. aureus*. In addition, all genes associated with the pathogenicity of *S. aureus* were inhibited, with the EA and EA-AuNPs showing a suppression rate of 99.9–100% compared to the control.

## 3. Discussion

Biofilms formed by microorganisms are a problem in various industries, such as food and medicine. Typically, antibiotics have been used to control biofilms, but the problem is that many resistant strains have emerged. Consequently, extensive research is being conducted to explore natural substances as alternatives to antibiotics for biofilm control. Therefore, this study conducted antibiofilm research by synthesizing nanoparticles using *E. bicyclis*, a brown alga with excellent bioactive substances, to enhance their stability.

Analysis of the physiologically active substances in *E. bicyclis* EA revealed the presence of phloroglucinol by GC-MS and various phlorotannins by LC-MS/MS, consistent with previous reports [31]. While these compounds demonstrate significant bioactivity, their inherent instability necessitates stabilization through nanoparticle synthesis, with EA serving as both a reducing agent and capping material [27]. The synthesized EA-AuNPs had an absorbance of 590 nm, a size of 165.04 ± 7.82 nm, and a stability value of −29.86 ± 2.91 mV (Figure 4). The size of the EA-AuNPs also falls within the nanometer scale range (1 nm to 1 µm), confirming their classification as nanoparticles [32]. Generally, nanoparticles exhibit high instability at zeta potentials between ±0 to 10 mV, moderate stability at ±10 to 20 mV, and become progressively more stable within the range of ±20 to 30 mV, reaching high levels of stability at values beyond ±30 mV [33]. To confirm the structural characteristics of EA-AuNPs, FT-IR and XRD analyses were performed to confirm that EA and AuNPs were synthesized successfully (Figure 5). Numerous studies have documented that Au(III) ions can be reduced to Au(0) through the action of -OH groups present in algal polysaccharides and polyphenols [34,35]. Additionally, the interaction between Au⁺ ions and EA metabolites was marked by a decrease in relative intensities, suggesting that EA acted as a capping agent, stabilizing the nanoparticles, a trend that aligns with previous findings [26]. The spherical morphology of EA-AuNPs was confirmed using FE-TEM, SEM, and EDS analysis (Figure 6). Spherical structures are commonly observed in AuNPs, and their formation is influenced by reaction conditions such as pH, temperature, and reaction time. Previous studies have reported that the morphology of AuNPs varies with pH, with rod-shaped structures formed at pH 2–4 and 10, spherical shapes at pH 8–9, and nanowires at pH 11 [36]. The antimicrobial results indicate that the *E. bicyclis* solvent-soluble fractions exhibit higher antibacterial activity against *S. aureus* than *P. aeruginosa*, with EA showing the highest activity (Table 1). Similarly, Kim et al. [30] previously reported strong antibacterial activity of the EtOAc fraction against streptomycin-resistant *Listeria monocytogenes*. Another study further demonstrated that AuNPs synthesized using the extract of brown alga *Ecklonia cava* have antibacterial activity against *Escherichia coli*, *Bacillus subtilis*, *P. aeruginosa*, and *S. aureus* [37]. These findings show that phlorotannins, found at high concentrations in EA, play a significant role in antibacterial activity. The aromatic ring and the -OH group of the phloroglucinol monomer were previously demonstrated to limit the growth of bacteria by interacting with bacterial proteins through hydrophobic interactions and hydrogen bonding [38]. EA-AuNPs exhibit antibacterial activity against both Gram-positive and Gram-negative bacteria. This efficacy is attributed to their ability to disrupt bacterial membranes, increase permeability, and induce oxidative stress, effectively inhibiting microbial growth [39]. These mechanisms highlight the potential of EA-AuNPs as broad-spectrum antimicrobial agents. Among the tested *E. bicyclis* solvent-soluble fractions, EA exhibited the highest biofilm inhibitory activity against *P. aerugionsa* and *S. aureus*, aligning with the MIC results. EA-AuNPs also demonstrated notable inhibition, particularly against *S. aureus*, achieving the highest biofilm inhibition levels (Figure 2). *Rhodiola rosea*-derived AuNPs were also shown to effectively inhibit biofilm formation by *P. aeruginosa* and *E. coli* at concentrations of 6.25 μg/mL and above [40]. Although brown algae-derived nanoparticles have been extensively studied primarily for their antioxidant, anticancer, and antibacterial properties, research on their potential for biofilm control has been limited [41]. In addition, biofilm inhibition activity was visually confirmed through SEM analysis (Figure S2). The SEM findings further indicated that AuNPs effectively interfered with bacterial colonization and aggregation. The findings of this study highlight the potential of EA and EA-AuNPs as effective inhibitors of biofilms formed by a wide range of pathogenic microorganisms, including Gram-positive and Gram-negative bacteria. Unlike most existing antibiofilm studies, which often focus on a single bacterial type, this research underscores the versatility of seaweed-derived nanoparticles. The CCK-8 assay revealed that EA-AuNPs exhibited significantly lower cytotoxicity compared to EA, highlighting their safety as an alternative form of *E. bicyclis* extracts. Additionally, previous studies have shown that bare AuNPs exhibit a 60% cell viability at concentrations ranging from 0.25 to 4 nM, whereas EA-AuNPs demonstrated enhanced biocompatibility, maintaining cell viability above 80% at comparable or higher concentrations [42]. This underscores the improved safety profile of EA-AuNPs for potential biomedical applications. According to ISO 10993-5, cell viabilities greater than 80% are considered non-cytotoxic. On the other hand, viabilities between 80% and 60% are considered to be mildly cytotoxic, between 60% and 40% are moderately cytotoxic, and above 40% are strongly cytotoxic [43].

Analysis of the motility inhibition activity of *P. aeruginosa* flagella confirmed that swimming and twitching were inhibited by EA and EA-AuNPs (Figure 7). Swimming motility, which is defined as the movement of individual cells in liquid that is propelled by rotating flagella, was greatly reduced. Flagellar and pili movements are crucial for surface attachment during the initial stages of *P. aeruginosa* biofilm formation. Our findings indicate that EA and EA-AuNPs effectively inhibited these early-stage mechanisms, thereby demonstrating their potential to control the onset of biofilm formation. EA and EA-AuNPs also inhibited pyoverdine, pyocyanin, and protease, which are virulence factors produced by *P. aeruginosa* (Figure 8). Pyoverdine is a siderophore and a key virulence factor that facilitates iron acquisition from the host environment. Pyocyanin, another critical virulence factor, functions as a QS signaling molecule and contributes to *P. aeruginosa* pathogenicity. Proteases are essential virulence factors in *P. aeruginosa* that damage host tissues and disrupt the innate antibacterial defenses of the host. These findings highlight the potential of EA and EA-AuNPs for the attenuation of multiple virulence factors in *P. aeruginosa*, including pyoverdine, pyocyanin, and protease. The inhibitory activity of staphyloxanthin, a virulence factor of *S. aureus*, was identified by color, and EA-AuNPs exhibited relatively high inhibition (Figure 9). Staphyloxanthin is a carotenoid pigment with a bright golden color. Staphyloxanthin is considered an important virulence factor of *S. aureus* due to its antioxidant properties. These results confirmed that EA-AuNPs exhibited superior inhibition of staphyloxanthin production compared with EA, highlighting their potential to attenuate the virulence of *S. aureus*. RT-PCR analysis results showed that EA and EA-AuNPs effectively inhibited the expression of genes involved in biofilm formation and virulence factor production in *P. aeruginosa* and *S. aureus* at sub-MIC levels (Figure 10). Natural extracts, such as those generated from brown algae, can block QS signaling and disrupt the early stages of biofilm growth and adherence [44]. In contrast, nanoparticles have been demonstrated to interfere with the expression of genes associated with QS, inhibit QS pathways, and reduce biofilm formation [45]. Nanoparticles can enter the biofilm matrix and exert their effects through physicochemical interactions, due to their small sizes. In addition, positively charged nanoparticles are particularly efficient against biofilms due to their greater capacity to penetrate the negatively charged matrix of biofilms [39]. Both EA and EA-AuNPs downregulated the expression of biofilm-related QS genes. This was demonstrated by the utilization of RT-PCR analysis as the method of investigation.

This study focused on biofilm formation under controlled laboratory conditions, which may not fully replicate the complexity of natural environments. Furthermore, the experiments were conducted using only two representative bacterial strains, *S. aureus* and *P. aeruginosa*, potentially limiting the generalizability of the findings. Future studies should incorporate more diverse microbial species and clinically relevant models to validate and expand upon these results. In our previous study, EB-AuNPs were synthesized using the MeOH extract of *E. bicyclis*, demonstrating significant antibacterial and antibiofilm activities. However, challenges related to stability and activity optimization remained. This study used EA, a fraction with higher phloroglucinol content, to synthesize EA-AuNPs. This approach resulted in nanoparticles with enhanced bioactivity, particularly more potent inhibition of virulence factors compared to EB-AuNPs [24]. These findings highlight the advantages of EA over crude extracts in nanoparticle synthesis for antimicrobial applications. Despite these limitations, this research provides valuable insights into the potential application of nanoparticles as biofilm inhibitors and lays a foundation for further exploration.

## 4. Materials and Methods

### 4.1. Preparation of E. bicyclis Methanolic Extract and Its Solvent-Soluble Fractions

Dried *E. bicyclis* was purchased from Ulleungdo Mall (Ulleungdo, Korea), ground to a size below 40 mesh, and stored at −20 °C until further use. Extraction of 1 kg of *E. bicyclis* was performed twice at 60 °C using 6 L of methanol (MeOH). Subsequent liquid–liquid extraction with *E. bicyclis* MeOH extract was carried out based on the polarity of the solvents [46]. After evaporation of the MeOH extract, the samples were first dissolved in water and then in *n*-hexane, dichloromethane (DCM), ethyl acetate (EtOAc), and *n*-butanol (BuOH), and extraction was performed in that order (Figure S1). All the solvents were purchased from Daejung Chemical & Metals Co., Ltd., Seoul, Korea. All solvents were removed using a rotary evaporator, and the resulting extracts were used for the subsequent experiments.

### 4.2. GC-MS Analysis

A gas chromatography–mass spectroscopy apparatus (GCMS QP-2010Ultra, Shimadzu, Kyoto, Japan) was used for metabolite analysis of the EtOAc fraction of *E. bicyclis*. Electron ionization was performed at 70 eV, and the mass range was between 40 and 600 *m*/*z*. For the gas chromatography, a DB-5MS Ultra insert column with 30 mm × 0.25 mm × 0.25 μm film thickness was purchased from Agilent Technologies (Santa Clara, CA, USA). Helium was used as the carrier gas, and the flow rate was maintained at 1.0 mL/min. Both the split ratio and the injector temperature were kept at 280 °C, with the split ratio being 1:100. The temperature of the column oven was kept at 60 °C for 2 min, and then gradually raised to 320 °C at a rate of 5 °C per minute for 20 min. Mass spectrum data were collected using the Wiley and National Institute of Standards and Technology (NIST) libraries.

### 4.3. LC-MS/MS Analysis

Phlorotannins in the *E. bicyclis* EtOAc fraction were identified using liquid chromatography–mass spectrometry/mass spectrometry (LC-MS/MS) using a C18 column (150 mm × 4.6 μm × 5 μm, Agilent Technologies, Santa Clara, CA, USA). Gradient elution was carried out using a solvent containing water, 0.1% formic acid, and acetonitrile, at a flow rate of 0.3 mL/min. Individual phlorotannins were tracked and identified using electrospray ionization mass spectrometry in the positive ion mode.

### 4.4. Synthesis of EA-AuNPs

The synthesis of AuNPs was performed according to a previously established procedure [24]. To ensure that the EA was completely dissolved, the extract was first dissolved in deionized water to a 25% (*w*/*v*) concentration and then thoroughly vortexed. The supernatant was collected for synthesis after centrifugation at 10,000 rpm for 10 min. A solution of 1 mM gold (III) chloride trihydrate (Sigma-Aldrich Company, St. Louis, MO, USA) was produced in deionized water, and the pH of the solution was adjusted to 9.0 by adding sodium hydroxide. The EA supernatant was then added dropwise to the gold solution. Completion of the synthesis was indicated by the development of a color similar to that of dark red wine. A spectrophotometer was used to record the absorbance spectra of the EA-AuNPs over a range of 200–700 nm at intervals of 10 min. This was performed to verify the success of the synthesis.

### 4.5. Microorganism Cultivation Conditions

Pathogenic and biofilm-forming Gram-positive *S. aureus* KCTC 1916 and Gram-negative *P. aeruginosa* PAO1 KCTC 1637 were chosen for the experiment. Tryptic soy broth (TSB; Difco Laboratory Inc., Detroit, MI, USA) was used to cultivate the bacteria. Each microorganism was cultured at 37 °C for 24 h and used in the experiment.

### 4.6. Determination of Minimum Inhibitory Concentrations

To determine the minimum inhibitory concentration (MIC), the broth microdilution method was applied using a 96-well microplate [47]. An inoculum density of 5 × 10^5^ CFU/mL was used to prepare the microbial cultures. Additionally, *E. bicyclis* solvent-soluble fractions and EA-AuNPs were subjected to a series of two-fold dilutions within the concentration range of 4–2048 µg/mL. The concentrations of EA and EA-AuNPs were determined by weighing the dried powder mass. Specifically, after complete drying, the powder was carefully weighed using an analytical balance to ensure accurate quantification. A control consisting of TSB was used to ensure the validity of the assay and to monitor for contamination. Plates were incubated at 37 °C for 24 h, and the optical density was measured at 600 nm with a Synergy HTX microplate reader (Biotek, Winooski, VT, USA). The MIC was determined as the lowest concentration of the test substances that completely inhibited the growth of visible microorganisms.

### 4.7. Biofilm Inhibition Assay

A biofilm inhibition assay was developed to investigate the biofilm inhibition activities of the synthesized AuNPs with *E. bicyclis* solvent-soluble fractions. Bacterial cultures were diluted to achieve an optical density (OD) value of 0.05 at 600 nm. A two-fold serial dilution approach was used to prepare *E. bicyclis* fractions and synthesize AuNPs. The concentrations of the samples ranged from 4 to 2048 μg/mL, with untreated samples serving as the control. Following dilution, the samples were distributed into 96-well plates and then incubated at 37 °C for 24 h. The medium was subsequently removed, and the wells were gently rinsed twice with distilled water before being allowed to air-dry for 30 min. Biofilm staining was achieved by adding 300 µL of 1% crystal violet solution to each well and incubating for 30 min. Immediately after staining, the crystal violet solution was withdrawn, and the wells were gently cleaned. A microplate reader was used to measure the amount of biofilm in each well with 300 µL of ethanol. The absorbance was then measured at 570 nm. The level of biofilm inhibition was calculated using the following formula:

Biofilm inhibition (%) = (OD of untreated sample) − (OD of treated sample)/OD of untreated sample × 100

### 4.8. SEM Analysis of Biofilms

The structures of biofilms formed by *P. aeruginosa* and *S. aureus* cells after treatment with EA and EA-AuNPs were investigated using scanning electron microscopy (SEM). To achieve an optical density of approximately 0.05 at 600 nm, the microorganisms were cultivated for a long period, and then diluted. A 24-well microplate, equipped with a nylon filter membrane measuring 0.1 μm (0.5 cm × 0.5 cm), was used to transfer the samples. The Sub-MICs of EA and EA-AuNPs were applied to the microbial cultures within the wells. Untreated bacterial cultures served as controls. Each culture was incubated for a period of 24 h at 37 °C. After fixing biofilm cells with 2% formaldehyde and 2.5% glutaraldehyde overnight at 4 °C, the cells were washed thrice with phosphate-buffered saline (PBS) to eliminate any cells that were not associated with the biofilm. After being dehydrated with 50–100% ethanol, adherent biofilm samples were freeze-dried for a period of two hours. SEM (JSM-IT800SHL, JEOL, Tokyo, Japan) was used to examine the surface of the biofilm on the membrane filter.

### 4.9. Cell Cytotoxicity Assay

An MTT assay was used to determine the cytotoxicity of the nanoparticles. Dulbecco’s modified Eagle’s medium (DMEM) supplemented with 10% fetal bovine serum (FBS), 100 units/mL penicillin, and 0.1 mg/mL streptomycin was used to culture RAW 264.7 cells under conventional conditions at 37 °C in a 5% CO_2_ environment [43]. Approximately 264.7 RAW cells were seeded into 96-well plates at a density of 2 × 10^5^ cells/mL. The EA and EA-AuNPs were utilized in a pre-incubation process with the cells at doses ranging from 4 to 2048 μg/mL. MTT solution (0.5 mg/mL in DMEM) was added to each well after treatment had been carried out for 24 h, and then the wells were incubated for 4 h. The medium was then removed, dimethyl sulfoxide (DMSO) was added to dissolve the formazan crystals, and after 30 min, the absorbance was measured using a microplate reader at 540 nm.

### 4.10. Characterization of EA-AuNPs

The synthesized EA-AuNPs were examined to determine their size, stability, structure, morphology, and spectral features. Dynamic light scattering (DLS; Litesizer 500, Anton Paar, Graz, Austria) was used to determine the size and zeta potential. Within the range of 4000 to 400 cm^−1^, the structural composition of the nanoparticles was determined using Fourier transform infrared (FTIR) spectroscopy (FT-4100, JASCO, Tokyo, Japan). X-ray diffraction (XRD; Ultima IV, Rigaku, Tokyo, Japan) was used to analyze the crystalline structure. Field emission transmission electron microscopy (FE-TEM; JEM-F200, JEOL, Tokyo, Japan) and energy-dispersive spectroscopy (EDS) mapping were used to better understand the morphology and elemental distribution of the EA-AuNPs.

### 4.11. Motility Test for P. aeruginosa

The motility of *P. aeruginosa* via the flagella, which manifests as swimming and twitching, was evaluated after treatment with EA and EA-AuNPs. For each assay, 3 μL of an overnight-grown *P. aeruginosa* culture was inoculated onto plates containing growth media. EA and EA-AuNPs were added to the media at final sub-MIC concentrations (1024 μg/mL and 512 μg/mL). The control plates were prepared with untreated samples.

The swimming assay medium comprised 0.25% NaCl, 1% tryptone, and 0.3% Bacto agar. The twitching assay medium comprised 0.2% casamino acids, 30 mM glucose, 1.5% Bacto agar, and LB broth. EA and EA-AuNPs were added to the swimming test fluid at various concentrations. After air-drying the media on a sterile bench, 3 μL of cultivated *P. aeruginosa* was inoculated into the center of the plate and allowed to dry. To perform the twitching experiment, 3 μL of *P. aeruginosa* culture was placed in the center of a sterile petri plate. Agar medium was placed on top and solidified after air-drying. After 24 h at 37 °C, motile cell diameter was measured and compared to the control in all plates. To conduct the twitching experiment, 3 μL of *P. aeruginosa* culture was placed in the center of a sterile petri plate. The firm medium was carefully layered on top when the inoculum was dried. After incubating all plates at 37 °C for 24 h, motile cell diameter was measured and compared to the untreated control. After incubation, the medium was removed, and the petri dish was stained with 1% crystal violet for 30 min for the twitching test. The diameters of the crystal violet-stained cells were measured after rinsing the plates with distilled water.

### 4.12. Assays for the Virulence Factors

An inhibitory assay was conducted to evaluate the effect of EA and EA-AuNPs on virulence factors associated with biofilm formation in *S. aureus* and *P. aeruginosa*. Staphyloxanthin pigment production was assessed as the primary virulence factor of *S. aureus*. Meanwhile, *P. aeruginosa*, a Gram-negative bacterium, was examined for its protease, pyocyanin, and pyoverdine production abilities. For each virulence factor assay, overnight cultures of the microorganisms were diluted in TSB to an OD at 600 nm (OD _600nm_) of 0.05. EA and EA-AuNPs were then introduced at concentrations below the sub-MIC level. In all the experiments, a negative control without treatment was included as the baseline, and each assay was performed in triplicate to ensure reproducibility and statistical reliability.

#### 4.12.1. Protease Activity Assay

To conduct the protease assay, cultures of *P. aeruginosa* that had been diluted previously were treated with either EA or EA-AuNP. The samples were shaken at a rate of 160 rpm while being incubated at 37 °C overnight. Following incubation, cultures were centrifuged at 10,000 rpm for 10 min. Subsequently, the supernatants were filtered using a 0.2-µm syringe filter obtained from ADVANTEC, Tokyo, Japan. While preparing the agar plates for the test, a medium containing 2% skim milk was used as a supplement. After that, 50 µL of the filtered supernatant was introduced into every well, and the mixture was then incubated at 37 °C for 24 h. To evaluate protease activity, the diameters of the clear zones surrounding each well were measured. The approach used to test the pyocyanin synthesis was identical to that employed in the protease experiment. After inoculation, the samples were incubated at 37 °C with shaking at 160 rpm for 12 h. Subsequently, 3 mL of chloroform was added to 5 mL of the culture sample, and the mixture was completely mixed. To extract pyocyanin, the chloroform layer was treated with 1 mL of hydrochloric acid at a 2% concentration and thoroughly mixed. During the extraction process, the color of the solution changed from pink to dark red, and the absorbance was measured at 520 nm to determine the amount of pyocyanin present.

#### 4.12.2. Pyocyanin Production Assay

Following inoculation, the samples were incubated at 37 °C and 160 rpm for 12 h. Subsequently, 3 mL of chloroform was added to 5 mL of the culture sample, and the mixture was completely mixed. To extract pyocyanin, the chloroform layer was treated with 1 mL of hydrochloric acid at a 2% concentration and thoroughly mixed. During the extraction process, the color of the solution changed from pink to dark red, and the absorbance was measured at 520 nm to determine the amount of pyocyanin present.

#### 4.12.3. Pyoverdine Production Assay

An overnight culture of *P. aeruginosa* grown in a minimum salt medium that included 2% sodium succinate was used to evaluate pyoverdine synthesis under the conditions described above. After inoculating the medium with EA and EA-AuNPs, the mixture was incubated at 37 °C for 12 h at a rotational speed of 160 rpm. Cultured samples were centrifuged at 10,000 rpm for 10 min to obtain the supernatant. To determine the amount of pyoverdine produced, the absorbance of the supernatant was measured at 405 nm.

#### 4.12.4. Staphyloxanthin Production

Staphyloxanthin, a potent antioxidant and key virulence factor, is produced by *S. aureus*, which is also responsible for producing gold carotenoid pigments. The EA- and EA-AuNP-treated and untreated *S. aureus* cells were incubated at 37 °C for 18 h at a rotational speed of 160 rpm. The samples were then centrifuged at 10,000 rpm for 10 min. The pellets were then washed twice with PBS. The cell pellets were photographed to confirm the production of staphyloxanthin. They were resuspended in 0.2 mL of MeOH to extract the carotenoid pigments and heated at 55 °C for 3 min. After centrifugation at 12,000 rpm for 10 min, the absorbance of the supernatant was measured at 465 nm.

### 4.13. RNA Isolation

Both *P. aeruginosa* and *S. aureus* cultures were diluted to OD_600_ of 0.05 and then subjected to treatment with sub-MIC concentration of EA and EA-AuNPs at 37 °C with overnight shaking. This was performed to isolate mRNA. Cells were collected by centrifugation at 13,000 rpm for 10 min. The obtained pellets were used to extract mRNA using the RiboEx^TM^ RNA extraction kit (RiboEx 301-001, GeneAll, Seoul, South Korea). A high-capacity complementary DNA (cDNA) synthesis kit (Applied Biosystems, Thermo Fisher Scientific, Waltham, MA, USA) was used to produce cDNA from the obtained mRNA. Reverse transcription polymerase chain reaction (RT-PCR) with SYBR^TM^ Green PCR master mix (Applied Biosystems) was performed using cDNA as a template.

### 4.14. RT-PCR Analysis of the Biofilm- and Virulence-Related Genes

The real-time PCR was used to investigate the expression of genes associated with biofilm formation, quorum sensing (QS) signaling, virulence factors, and motility in *P. aeruginosa* and *S. aureus*.

For *P. aeruginosa*, primers to amplify QS signaling genes (*rhlR-rhlI* and *lasR-lasI* systems), the exopolysaccharide gene involved in biofilm matrix formation, sigma factor (*algU*), genes associated with virulence (*phzC, phzE*, and *lasB*), and the flagellar gene (*flgG*), are listed in Table S1. The primers for the expression of bacterial genes in *S. aureus* targeted QS signaling genes (*argA* and *RNAIII*), two-component system genes (*arlR* and *arlS*), biofilm-related genes (*icaA* and *rbf*), and virulence- and toxin-associated genes (*hla*, *saeR*, *sarZ*, *sigB*, *spa*, *nuc1*, and *nuc2*). The primer sequences used for *S. aureus* analysis are presented in Table S2. Using the housekeeping genes *proC* and 16S rRNA, gene expression levels in *P. aeruginosa* and *S. aureus* were standardized. The 2^−ΔΔCT^ approach, as reported earlier, was utilized to ascertain the relative quantification of gene expression during the analysis [48].

### 4.15. Statistical Analysis

Each experiment was independently performed in triplicate. GraphPad Prism 7.0 (GraphPad Software Inc., San Diego, CA, USA) was used to analyze the data. One-way analysis of variance (ANOVA) was used to determine statistical significance. The *p*-values, marked *** *p* < 0.001, ** *p* < 0.01, and * *p* < 0.05, were used to indicate the degree of significance. Statistical Package for the Social Sciences (SPSS) version 27 (Chicago, IL, USA) was used for additional statistical analyses.

## 5. Conclusions

This study evaluated the antibacterial and antibiofilm activities of EA from the brown alga *E. bicyclis* and EA-AuNPs. Major phlorotannins, including eckol and dieckol, were identified as active compounds in EA. EA showed greater antibacterial and antibiofilm properties compared to EA-AuNPs. However, EA-AuNPs, characterized by improved stability and structure, also demonstrated reduced cytotoxicity, enhancing their overall safety profile. EA and EA-AuNPs significantly inhibited virulence factor production in *P. aeruginosa* and *S. aureus,* reducing pyoverdine, pyocyanin, protease, and staphyloxanthin synthesis. RT-PCR analysis confirmed the downregulation of biofilm- and virulence-related genes. To our knowledge, this is the first study to report the antibiofilm activity of EA-AuNPs against both Gram-positive and Gram-negative bacteria, demonstrating their unique potential to combat diverse pathogens. These findings highlight the promising applications of EA and EA-AuNPs in controlling pathogenic bacteria. Given their enhanced safety and stability compared to EA, EA-AuNPs are particularly well-suited for food safety and infection management applications. Future studies should explore their potential in clinical and industrial settings to further harness their versatility and efficacy.

## Figures and Tables

**Table 1:** MIC values of *Eisenia bicyclis* solvent-soluble fractions and *Eisenia bicyclis* ethyl acetate fraction conjugated with gold nanoparticles (EA-AuNPs) against *Pseudomonas aeruginosa* and *Staphylococcus aureus*.

Samples		*P. aeruginosa*	*S*. *aureus*
*E. bicyclis* fractions	MeOH	>2048 μg/mL	1024 μg/mL
	BuOH	>2048 μg/mL	>2048 μg/mL
	DCM	>2048 μg/mL	256 μg/mL
	EtOAc	1024 μg/mL	128 μg/mL
	*n*-hexane	>2048 μg/mL	>2048 μg/mL
	Water	>2048 μg/mL	>2048 μg/mL
EA-AuNPs		2048 μg/mL	256 μg/mL

MeOH, methanol; BuOH, *n*-butanol; DCM, dichloromethane; EtOAc, ethyl acetate.

## Data Availability

No datasets were generated or analyzed during the current study.

## Supplementary Materials

The following supporting information can be downloaded at https://www.mdpi.com/article/10.3390/antibiotics14020182/s1, Table S1: Sequence of RT-PCR primers for *Pseudomonas aeruginosa*; Table S2: Sequence of RT-PCR primers for *Staphylococcus aureus*; Table S3: GC-MS profiling of bioactive compounds in *Eisenia bicyclis* ethyl acetate fraction; Table S4: Bioactive compounds identified in *Eisenia bicyclis* ethyl acetate fraction by LC-MS/MS analysis; Figure S1: Schematic diagram showing liquid–liquid extraction of *Eisenia bicyclis* methanolic extract; Figure S2: Biofilm cells of *Pseudomonas aeruginosa* and *Staphylococcus aureus* treated with *Eisenia bicyclis* ethyl acetate fraction (EA) and EA conjugated with gold nanoparticles (EA-AuNPs). (A) *P. aeruginosa* control biofilm cells. (B) *P. aeruginosa* biofilm treated with EA. (C) *P. aeruginosa* biofilm treated with EA-AuNPs. (D) *S. aureus* control biofilm cells. (E) *S. aureus* biofilm treated with EA. (F) *S. aureus* biofilm treated with EA-AuNPs.

## Abbreviations

The following abbreviations are used in this manuscript:

ACTC	American Type Culture Collection
AMR	antimicrobial resistance
AuNPs	gold nanoparticles
CFU	colony forming unit
EDS	energy dispersive spectroscopy
EA	*Eiseina bicyclis* ethyl acetate fraction
FE-TEM	field emission transmission electron microscopy
FTIR	Fourier transform infrared spectrometer
KCTC	Korean Collection for Type Cultures
NPs	nanoparticles
MIC	minimum inhibitory concentration
SAED	selected area electron diffraction
SEM	scanning electron microscopy
TSB	tryptic soy broth
XRD	X-ray diffractometer
