# Supplementary material for: Eisenia bicyclis-Mediated Gold Nanoparticles Exhibit Antibiofilm and Antivirulence Activities Against Pseudomonas aeruginosa and Staphylococcus aureus
Source: Antibiotics (Basel). 2025 Feb 11;14(2):182. doi: 10.3390/antibiotics14020182 (PMC11851435; doi:10.3390/antibiotics14020182)
Supplement: Supplementary file 1 [file antibiotics-14-00182-s001.zip › antibiotics-3452395-supplementary.pdf]

Table S1. Sequence of RT-PCR primers for *Pseudomonas aeruginosa*

Gene	Function	Sequences (5′ to 3′)
algA	Biofilm-associated genes	F: AGAACTGAAGAAGCACGACG<br>R: TTCTCCATCACCGCGTAGT
algU	Sigma factor	F: AACACCGCGAAGAACCACCT<br>R: ATCTCATCCCGCAACATCGCG
pleA	Biofilm-associated genes	F: ATGGCTGAAGGTATGGCTG<br>R: AGGTGCTGGAGGACTTCATC
lasB	QS regulated genes	F: GACAACGCGTCGCAGTA<br>R: AGGTAGAACGCACGGTTGTACA
lasI	QS regulatory gene	F: GCCCCTACATGCTGAAGAACA<br>R: CGAGCAAGGCGCTTCCT
lasR	QS regulatory gene	F: GACCAGTTGGGAGATATCGGTTA<br>R: TCCGCCGAATATTTCCCATA
rhlI	QS regulatory gene	F: GCAGCTGGCGATGAAGATATTC<br>R: CGAACGAAATAGCGCTCCAT
phzC	Pyocyanin phenazine biosynthesis protein	F: AGCGGATCCTCAAGGGCTAT<br>R: GTGGGTCGAACCGAGATAGA
phzE	Phenazine biosynthesis protein	F: CTCGACAACCGCAAGGAA<br>R: ATCCGCGCCATCATCTTC
flgG	Flagellar basal body rod protein	F: CACTGTGGGTCAGCAAGACC<br>R: TACAGCAGGTCCTGGAACTC
proC	Housekeeping gene	F: CAGGCCGGGCAGTTGCTGTC<br>R: GGTCAGGCGCGAGGCTGTCT

Table S2. Sequence of RT-PCR primers for *Staphylococcus aureus*

Gene	Functions	Sequences (5′ to 3′)
agrA	Quorum-sensing regulator A	F: TGATAATCCTTATGAGGTGCT<br>R: CACTGTGACTCGTAACGAAAA
arlR	Response regulator	F: TTACGGTGCAGGCGATTATATAG<br>R: TACCGTTGACATCGATAATATCC
arlS	Histidine-protein kinase	F: TGGAATACCAATTCCATGATCT<br>R: TGCAATCAAATATGATGTGAAGAA
aur	Zinc metallo-proteinase aureolysin	F: ACCGTGTGTTAATTCGTGTGCTA<br>R: ATGGTCGCACATTCACAAGTTT
hla	α -Hemolysin	F: CGGCACATTTGCACCAATAAGGC<br>R: GGTTTAGCCTGGCCTTCAGC
icaA	Intercellular adhesion A	F: TGAACCGCTTGCCATGTG<br>R: CACGCGTTGCTTCCAAAGA
nuc1	Nuclease	F: CACCTGAAACAAAGCATCCTAA<br>R: TATACGCTAAGCCACGTCCAT
nuc2	Nuclease	F: ATGGACGTGGCTTAGCGTAT<br>R: TGACCTGAATCAGCGTTGTC
rbf	Regulator of biofilm formation	F: TTAGAAGGAATCTTTAAAACCTTATTGAATAA<br>R: TTGTGAATTTTTCTTCTTCGGACA
RNAIII	Transcriptional regulator	F: ATCGACACAGTGAACAAATTCAC<br>R: CTCTACTAGCAAATGT TACTCAC
saeR	Response regulator	F: GCCTTAACTTTAGGTGCAGATGACTATGTC<br>R: CGACAGTTGTTCAACTGGTTGATGATGG
sarZ	HTH-type transcriptional Regulator	F: CCTATACTGGTTACATTGTTTTAATGG<br>R: TGGTGTCAGTGTTCCAGAATC
sigB	RNA Polymerase sigma factor	F: AAGTGATTCGTAAGGACGTCT<br>R: TCGATAACTATAACCAAAGCCT
spa	Protein A	F: ACCAGAAACTGGTGAAGAAAATCC<br>R: TAACGCTGCACCTAAGGCTAATG
16*S rRNA*	A component of ribosomes	F: TGTTTGACGATGTTTGAGCA<br>R: CCTTCCTCCAGTTCAGATGC

Table S3. GC-MS Profiling of bioactive compounds in *Eisenia bicyclis* ethyl-acetate fraction

Peak No.	Compound name	Molecular formula	Retention (RT) time	Peak area (%)	Similarity percentage (%)	Chemical structure
1	Succinic anhydride	$C_4H_4O_3$	6.58	5.11	93	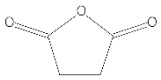
2	Monomethyl succinate	$C_5H_8O_4$	8.00	12.24	96	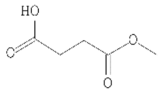
3	Phloroglucinol	$C_6H_6O_3$	15.30	78.58	97	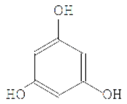
4	Neophytadiene	$C_{20}H_{38}$	18.48	1.22	93	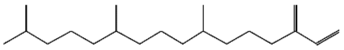
5	Unknown	-	19.69	0.88	-	-
6	Palmitic acid	$C_{16}H_{32}O_2$	20.15	1.97	95	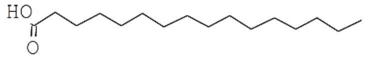

Table S4. Bioactive compounds identified in *Eisenia bicyclis* ethyl-acetate fraction LC-MS/MS Analysis

<table>
<tr><th>Compound</th><th>Molecular Formula</th><th>Molecular weight</th><th>Target molecular weight (positive mode,m/z,z=1)</th><th>Result (m/z)</th></tr>
<tr><td>Eckol</td><td>$C_{18}H_{12}O_9$</td><td>372.3 g/mol</td><td>373.0556 g/mol</td><td>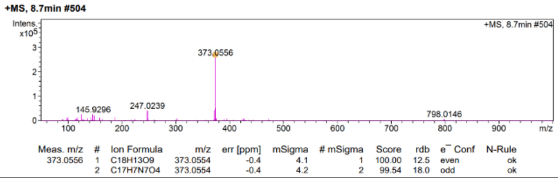
</td></tr>
<tr><td>Dieckol</td><td>$C_{36}H_{22}O_{18}$</td><td>742.5 g/mol</td><td>743.0887 g/mol</td><td>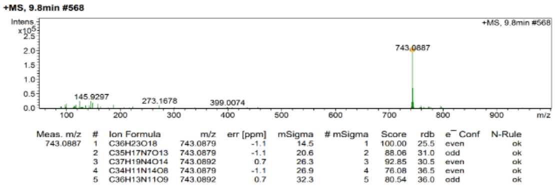
</td></tr>
<tr><td>6,6'-bieckol</td><td>$C_{36}H_{22}O_{18}$</td><td>742.5 g/mol</td><td>743.0875 g/mol</td><td>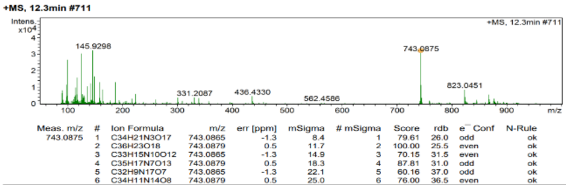
</td></tr>
<tr><td>Phlorofuco -furoeckol</td><td>$C_{30}H_{18}O_{14}$</td><td>602.5 g/mol</td><td>602.0767 g/mol</td><td>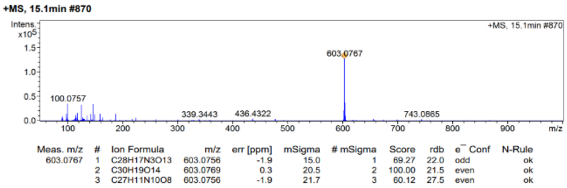
</td></tr>
</table>

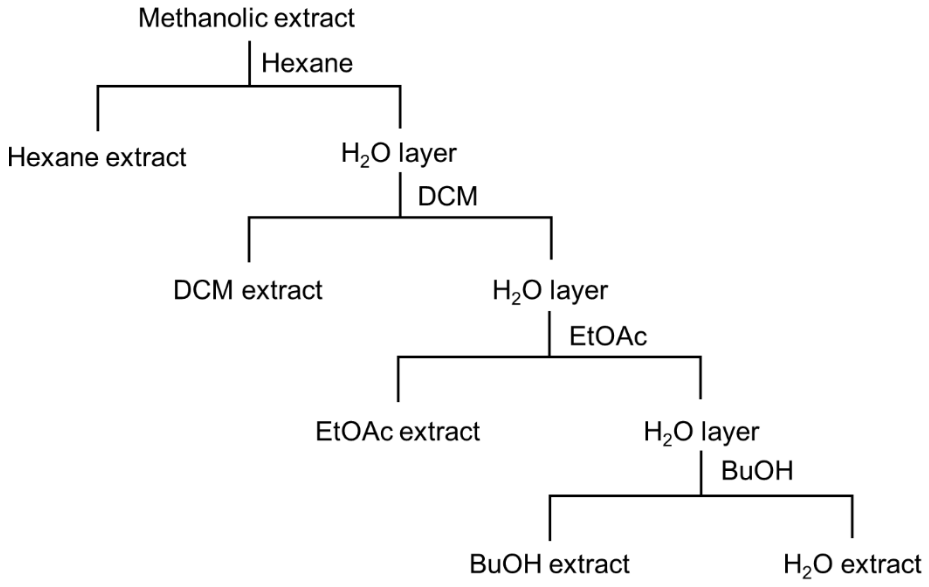

Figure S1. Schematic diagram showing liquid-liquid extraction of *Eisenia bicyclis* methanolic extract.

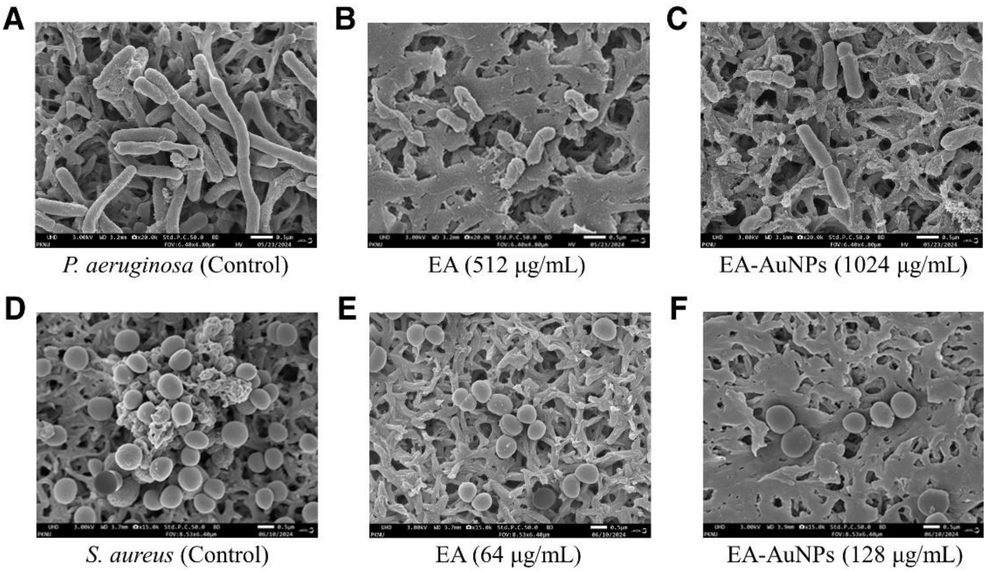

Figure S2. Biofilm cells of *Pseudomonas aeruginosa* and *Staphylococcus aureus* treated with *Eisenia bicyclis* ethyl-acetate fraction (EA) and EA conjugated with gold nanoparticles (EA-AuNPs). **(A)** *P. aeruginosa* control biofilm cells, **(B)** *P. aeruginosa* biofilm treated with EA, **(C)** *P. aeruginosa* biofilm treated with EA-AuNPs, **(D)** *S. aureus* control biofilm cells, **(E)** *S. aureus* biofilm treated with EA, and **(F)** *S. aureus* biofilm treated with EA-AuNPs.
